# The Kessler psychological distress scale: translation and validation of an Arabic version

**DOI:** 10.1186/s12955-017-0783-9

**Published:** 2017-10-27

**Authors:** Scott D. Easton, Najwa S. Safadi, Yihan Wang, Robert G. Hasson

**Affiliations:** 1 School of Social Work, Boston College, 140 Commonwealth Avenue, McGuinn Hall, Room 207, Chestnut Hill, MA 02467 USA; 2 Department of Social Work, Al-Quds University, Main Campus, Abu Dies, P.O Box 51000, Jerusalem, Palestine

**Keywords:** Kessler psychological distress scale, Palestine, Occupied Palestinian territories, Arabic, Mental distress, Confirmatory factor analysis

## Abstract

**Background:**

The Kessler Psychological Distress Scale has been widely used in assessing psychological distress among general and clinical populations from different cultural backgrounds. To our knowledge, however, researchers have not yet validated any translated versions in Arabic. The purpose of this study was to test the reliability and validity of Arabic translations of the ten item (K10) and six item (K6) versions among public sector employees in the Occupied Palestinian Territories.

**Methods:**

As part of a larger research project on life satisfaction, researchers collected data from 234 Palestinian social workers in June and July of 2016. The survey included several mental health measures, including the K10, which were translated from English to Arabic by an experienced language expert. In the current study, we tested reliability by measuring internal consistency using Cronbach’s alpha coefficient. Next, we assessed factor structure using variance-covariance matrix with maximum likelihood estimation. Confirmatory factor analysis was performed to examine three competing models: unidimensional K10 model, unidimensional K6 model and two-factor K6 model. Fit indices and parameter estimates were reported. Last, convergent validity was examined by assessing correlations with Generalized Anxiety Disorder (GAD-7) and Somatic Symptoms Scale (SSS-8).

**Results:**

The mean scores for the K6 and K10 were, respectively, 12.87 (*SD* = 4.02) and 21.8 (*SD* = 6.7), indicative of mild to moderate levels of distress. Scale reliability analysis showed satisfactory results on both K6 and K10 versions (Cronbach’s α = .81 (K6) and .88 (K10)). Among three competing models, the two-factor K6 scale demonstrated the best model fit with high factorial correlations (*r* = .60, *p* < .001). Moreover, the K6 has high convergent validity with GAD-7 (*r* = .66, *p* < .001) and SSS-8 (*r* = .61, *p* < .001).

**Conclusion:**

Results indicated that the translated version of the two-factor K6 scale is a valid and reliable measurement of psychological distress. Our findings suggest that practitioners and researchers can use this instrument in screening and assessing psychological symptoms with Arabic-speaking populations.

## Background

The Kessler Psychological Distress Scale (K10) is a well-validated, highly useful clinical measure of psychological symptoms noted for its ease of use, accessibility, high predictability, and high factorial and construct validity [1, 2]. The six-item version (K6) is a well-validated adaptation [3]. Both versions have been used to assess psychological distress across multiple settings and populations including military personnel [4], private sector employees [5], adults living with diabetes [6], adolescents [7], and older adults [8].

K10 has been validated with diverse populations from Australia [9], South Africa [10], France [11], New Zealand [12], Hong Kong [7], and American Indian communities [13]. The instrument has also been validated for use in languages other than English including Korean [8], Mandarin [7], French [11], Spanish [14], Dutch [15], and Turkish [15]. Although the K10 has been validated with diverse populations and in different languages, research suggests there are discrepancies in the factor structure of the K6 and K10 scales. For example, Bessaha [3] highlighted differences between one–factor and two–factor structures of the K6, and Brooks, Beard, and Steel [16] identified differences between four –factor and two–factor structures of the K10. These discrepancies indicate that more research on psychometric properties of the K10 and K6 instruments is warranted.

Despite the wide use of this instrument including some translated versions in Arabic [17, 18], our literature review did not identify any empirical validation studies with Arabic-speaking populations. This is surprising considering there are an estimated 392 million people who inhabit the 22 Arabic-speaking countries in the world [19]. One such nation, the Occupied Palestinian Territories (OPT), presents a unique and important context for validating these measures. Environmental conditions such as fragile structure of government, military occupation, and high rates of social problems e.g., food insecurity, poverty, unemployment; [20, 21] constitute formidable challenges for developing anti-poverty programs [22] and threaten the mental health of residents. Thus, the purpose of this study was to assess the psychometric properties—internal consistency, factor analysis, and test validity-- of an Arabic translation of both versions of the Kessler Psychological Distress Scale. Validation of these instruments would enhance our ability to accurately measure mental distress among Palestinians in OPT and, potentially, Arabic-speaking individuals around the globe.

## Methods

### Design

The current study is based on data collected in June and July of 2016 as part of a larger investigation into life satisfaction among public sector social workers in the OPT. The investigation used a cross-sectional design and employed convenience sampling techniques. The project received support from the Ministry of Social Development (MOSD; formerly Ministry of Social Affairs) of the Palestinian National Authority and human subjects approval from the Institutional Review Board at a major research university in the Northeast of the United States of America. As such, it was deemed to be in compliance with ethical standards for research, including the Declaration of Helsinki.

### Data source

The target population consisted of MOSD social workers who are organized into 12 directorates and local offices in West Bank cities and towns such as Ramallah, Jericho, Salfit, Nablus, and Hebron. These public employees provide a wide range of direct services (e.g., economic assistance, health prevention/treatment, educational and social programming) to various constituents: abused children, disabled individuals with chronic conditions, older adults, battered women, and families and individuals living in poverty.

Researchers worked with MOSD administrators to develop a schedule for data collection and sent an announcement of the voluntary, unpaid research opportunity to each local office. The second author then visited directorates and local offices, holding small group meetings to introduce the purpose and procedure of the study and distribute and review consent forms. Interested participants signed consent forms prior to completing the survey. The researcher remained on-site to answer questions, collect surveys, and debrief participants.

The survey consisted of 100 closed-ended items based on adapted versions of standardized measures of concepts such as life satisfaction, organizational support, job stress, and mental and physical well-being. Measures of mental and somatic health were situated within the first one-third of questions in the survey; demographic and background questions were contained in the last one-third of the survey. Measures were translated from English into Modern Standard Arabic by a nationally-certified Arabic language instructor with nearly three decades of teaching experience at the high school and college levels in the United States and the Middle East. She is a leader in the design of Arabic language curriculum for both traditional and online courses at U.S. colleges and high schools and has nearly 20 years of professional translation experience, including standardized state educational assessments such as the Michigan Educational Assessment Program.

To promote accuracy, standard translation protocol and techniques (e.g., adaption, transposition, multiple sourcing; [23]) were employed. Additionally, two faculty members at Al-Quds University, Jerusalem, completed quality checks for the entire translated survey. Both professors have appointments in the Department of English Language and Literature, hold doctorate degrees, and have research expertise in translator training, translation technology, and discourse analysis. Quality checks resulted in numerous clarifications and modifications to ensure items were comprehensive and acceptable to the target group. Researchers kept extensive records of translation efforts as part of a thorough audit trail.

### Measures

#### Psychological distress

This concept was assessed using the Kessler Psychological Distress Scale, a measure of non-specific psychological distress based on a framework that includes behavioral, emotional, cognitive, and psychophysiological manifestations [2]. The scale was created using highly sensitive items that identify extreme psychological distress in the general population. The ten-item version (K10) measures frequency with which respondents experienced symptoms in the past month, including nervousness, hopelessness, sadness, worthlessness, and fatigue. Response choices are based on 5-point Likert-type scale ranging from 1 (*none of the time*) to 5 (*all of the time*). Responses are summed to create a total score (range = 10–50) with higher scores signifying more psychological distress. Research has suggested that the optimal cut-point for a psychological disorder is 24 [24]. In previous studies, K10 had strong scale reliability with Cronbach’s α greater than 0.88 [15, 25].

K6 is a shortened, six-item version of the K10 that assesses frequency of the following mental health symptoms in the past month: feeling nervous, hopeless, restless or fidgety, so sad that nothing could cheer them up, that everything was an effort, and worthless. In the current study, items were extracted from the K10 and used the same response set. [9, 26]. Responses were summed to produce a total score (range = 6–30), with higher scores signifying more distress. Based on a previous study [13], the K6 cutoff point for psychological disorders for our study was 16.25. K6 has been found to be reliable with Cronbach’s α ranging from 0.89 to 0.92 [1].

Both scales are easy to understand and publicly available; interviewer-administration and self-administration versions are online [1]. English versions of K10 [16] and K6 [3, 27] have been validated by past research.

#### Generalized anxiety

Generalized Anxiety Disorder (GAD-7) is a seven-item measure of respondents’ level of recent anxiety [26]. Respondents were asked how often they were bothered by problems (e.g., “not being able to stop or control worrying” or “worrying too much about different things”) in the past two weeks. Response choices were based on a 4-point Likert-type scale ranging from 0 (*not at all*) to 3 (*nearly every day*). Item responses were summed to produce a total score ranging from 0 to 21; higher scores signified more anxiety. Previous research among patients in primary care clinics suggested a cut point score of 10 for identifying anxiety disorders [26].

#### Somatic symptoms

Somatic Symptoms Scale (SSS-8) was used to assess the level of recent somatic symptoms burden. Previous research has found that the SSS-8 is a reliable and valid self-report measure of somatic symptom burden [28]. Respondents were asked how often in the past week they were bothered by common problems such as headaches, pain (arm/leg/joint), stomach or bowel problems, and sleep problems. Response choices were based on a 5-point, Likert-type scale ranging from 0 (*not at all*) to 4 (*very much*). Total scores ranged from 0 to 32, with higher scores signifying more burdens. Suggested cut points for SSS-8 are as follows: 0–3 points (minimum to no burden), 4–7 points (low), 8–11 points (medium), 12–15 points (high), over 16 points (very high burden) [28].

#### Background characteristics

Demographic and background characteristics were assessed, including age (*years*), gender (*male/female*), marital status (*married, never married, other*), educational level (*secondary diploma, college diploma, bachelor’s degree, master’s degree or higher*) refugee status (*yes/no*), full-time employment (*yes/no*), and monthly income (*U.S. dollars*).

### Data analysis

Descriptive statistics and correlation tests were performed using SPSS, version 24.0 [29]. Confirmatory factor analysis (CFA) was performed using LISREL, version 9.1 student edition [30]. Consistent with recommended practice when a dataset has minimal levels of missing data (i.e., < 5%), listwise deletion was used [31]. Cases with missing data on variables of interest in our analysis were removed, resulting in a final sample size of 234. Before reporting univariate statistics for demographic background and mental health variables, multivariate normality was examined and confirmed for both K6 and K10 versions.

Next, a variance-covariance matrix with maximum likelihood (ML) estimation was used as input matrix. We reported and compared model fit indices for three models: one-factor K10 model, one-factor K6 model, and two-factor K6 model. χ2 statistics and significance levels were reported. A large and significant χ2 indicates poor model fit [32]. As suggested by Schmitt [33], we went beyond a global model evaluation and conducted additional analysis using several fit indices: root mean square error of approximation (RMSEA; 34), comparative fit index (CFI; [34]), Akaike information criteria (AIC), Bayesian information criteria (BIC), and standardized root mean square residual (SRMR; [34]). We applied Byrne’s [32] suggestion that fit indices should serve as guidelines that provide information on a model’s lack of fit and should be used along with “theoretical, statistical, and practical considerations” (p. 77). Current guidelines suggest that CFI values greater than or equal to 0.90 indicate acceptable fit; values greater than or equal to 0.95 imply very good fit [35]. RMSEA values less than 0.05 indicate close model fit, and values exceeding 0.10 indicate poor fit [36]. SRMR values less than 0.08 also indicate good fit [35].

We also examined standardized residuals and individual parameter estimates for three models. In studies that screen for mental illness among the general population, Kessler et al. [27] suggested that the unidimensional K6 model performs the best. In another study that examined populations for non-specific psychological distress, Kessler and colleagues [1] found support for a single factor model of K10. Bessaha [3] suggested a two-factor K6 has better model fit than one-factor K6 in screening for psychological distress within young adult populations. To compare the three competing models among our sample, we examined each question on the scale, evaluating signs and magnitude of each parameter.

Last, we performed the Pearson’s correlation test to examine relationships between the K6 total score and its subscores. We also evaluated convergent validity by examining correlations between K6 and two other scales measuring mental health: GAD-7 and SSS-8.

## Results

### Descriptive statistics

Descriptive statistics for our sample are presented in Table 1. The mean age of our study sample (*N* = 234) was 38.16 years (*SD* = 9.76, range = 25–58). The majority of participants were female (70%), married (78.1%), and college-educated (84.4%). Most respondents did not self-identify as refugees (65.3%). A high percentage of respondents (84%) reported that they were employed full time. Mean monthly income from their job was $842 (*SD* = 210.84).

**Table 1:** Descriptive Statistics of Sample (*N* = 234)

	Mean (SD) / %
Demographic Background	
Age (years)	38.16 (9.76)
Gender (%)	
Male	30.3
Female	69.7
Marital Status (%)	
Married	78.1
Never married	16.0
Divorced/widowed/separated	5.9
Educational level (%)	
Secondary diploma	0.4
College diploma	3.8
BA degree	84.4
MA or higher	9.7
Refugee status (% yes)	34.7
Employed full time (% yes)	83.8
Individual monthly income^1^	842.13 (210.84)
Mental Health Measures	
K10	21.75 (6.72)
K6	12.87 (4.02)
GAD-7	5.37 (4.57)
SSS-8	12.22 (7.86)

Note: ^1^Income is measured in US dollars, converted from Israeli Shekels using XE Currency Convertor (1ILS = 0.274 USD) on 3/23/17

For mental health measures, the mean K10 score was 21.75 (*SD* = 6.72), which indicates moderate levels of distress [24]. The mean K6 score was 12.87 (*SD* = 4.02), indicative of mild distress [3]. The mean GAD-7 score was 5.37 (*SD* = 4.57), indicating relatively low scores on anxiety below the clinical cut point [26]. The mean score of SSS-8 was 12.22 (*SD* = 7.86), indicating high levels of somatic symptoms [28].

### Reliability

Results indicated that K10 had strong scale reliability with Cronbach’s α equal to 0.88. The scale reliability for K6 was good with Cronbach’s α equal to 0.81.

### Confirmatory factor analysis

Three separate CFA models were tested to compare fit indices of each factor structure and determine which model had the best fit for our data. Results are presented in Table 2. The χ2 statistics for the K10 one-factor model and K6 one-factor model were significant, which indicates poor fit. The χ2 statistics for K6 two-factor model was not significant, indicating good fit. All other model fit indices indicated that the two-factor K6 version had the best model fit. SRMR (.0244) and RMSEA (.040) were below the cutoff point of .05, suggesting satisfactory model fit. The value of CFI (.996) was excellent. Overall, the two-factor model of K6 had very good fit statistics and performed better than unidimensional models of either the K10 or K6.

**Table 2:** Confirmatory Factor Analysis Model Fit Indices

Model	χ2 *(*df*, p)*	RMSEA (90% CI)	CFI	AIC	BIC	SRMR	Δ χ2 *(*df*, p)*
K10 One-factor	179.579 (35, *p* < .01)	.135 (.115, .154)	.922	1400.213	1468.800	.0743	
K6 One-factor	60.022 (9, *p* < .01)	.156 (.120, .194)	.919	866.255	907.718	.0718	119.535 (26, *p* < .01)
K6 Two-factor	8.276 (6, *p* = 0.2186)	.040 (.00, .100)	.996	820.509	872.339	.0244	51.746 (3, *p* = 0.2186)

Notes: χ2 = chi-square; *df* = degrees of freedom; RMSEA = root mean square error of approximation; CI = confidence interval; CFI = comparative fit index; AIC = Akaike information criteria; BIC = Bayesian information criteria; SRMR = standardized root mean square residual

Standardized and unstandardized parameter estimates for each model are presented in Table 3. Factor loadings for each of the three models showed good fit to the data with statistically significant results on all loadings (*p* < .001). For the two-factor K6 model, we examined parameter estimates and found that signs and magnitudes were, as expected, greater than 0.55. Model modifications were not practiced.

**Table 3:** Standardized and Unstandardized Factor Loadings for Confirmatory Factor Analysis Models

Item	Kessler 10 One Factor		Kessler 6 One Factor		Kessler 6 Two Factors		
		SE		SE	Factor 1 Anxiety	Factor 2 Depression	SE
Did you feel tired for no good reason?	0.596 (1.000)*	–	–	–	–	–	–
Did you feel nervous?	0.609 (0.963)*	0.128	0.433 (1.000)*	–	0.574 (1.000)*	–	–
Did you feel so nervous that nothing could calm you down?	0.642 (1.022)*	0.131	–	–	–	–	–
Did you feel that everything was an effort?	0.627 (1.093)*	0.142	0.622 (1.586)*	0.281	–	0.730 (1.000)*	–
Did you feel so sad that nothing could cheer you up?	0.690 (1.137)*	0.138	0.716 (1.705)*	0.287	–	0.820 (1.048)*	0.125
Did you feel worthless?	0.615 (0.917)*	0.121	0.721 (1.556)*	0.261	–	0.625 (0.723)*	0.105
Did you feel restless or fidgety?	0.698 (1.098)*	0.132	0.629 (1.438)*	0.254	0.889 (1.530)*	–	0.239
Did you feel so restless that you could not sit still?	0.642 (1.098)*	0.152	–	–	–	–	–
Did you feel depressed?	0.754 (1.293)*	0.148	–	–	–	–	–
Did you feel hopeless?	0.620 (0.959)*	0.126	0.732 (1.638)*	0.273	–	0.644 (0.773)*	0.110

Notes. Unstandardized factor loading values are in parenthesis. SE = standardized error. *p < .001 for standardized factor loadings

### Convergent validity

Due to model results from CFA described earlier, we focused on the K6 in subsequent analysis. Table 4 provides inter-correlations of the K6 total score, K6 sub-scores, and other measures of psychological problems. Factor correlations were high (> 0.80). Convergent validity was also demonstrated, as correlations between K6 and other measures of psychological problems (i.e., GAD-7; SSS-8) were near or above 0.60.

**Table 4:** Inter-correlations of K6 score, K6 sub-scores, and Mental Distress Measures

	Kessler 6	Depression	Anxiety	GAD-7	Somatic
Kessler 6	1.00				
Depression	.95*	1.00			
Anxiety	.83*	.60*	1.00		
GAD-7	.66*	.56*	.53*	1.00	
Somatic	.61*	.52*	.60*	.63*	1.00

Note: Pearson correlation coefficients are shown. *p < .001(two-tail)

## Discussion

The Kessler Psychological Distress Scale is a well-known instrument for measuring non-specific symptoms of psychological problems that has been translated and validated into many languages [7, 8, 11, 14, 15]. However, our literature review did not identify any validation studies that assessed psychometric qualities of translated versions in the Arabic language. Based on a sample drawn from the Occupied Palestinian Territories, our study assessed the reliability and validity of two versions of this instrument (K6, K10) and found that the two-factor, K6 scale had generally promising results.

More specifically, internal consistency was high in our study for both versions and consistent with previous research [1, 15, 25]. These findings suggest that the translated items in fact measure the same overall construct of psychological distress. In terms of the dimensional structure of the instrument, there is some variation in results with different populations. Some research has supported use of the unidimensional K10 [1], a unidimensional K6 [27], and a two-factor K6 [3]. Results of the current study indicated that within a sample of Arabic-speaking sample from the OPT, the two-factor K6 model (depression and anxiety) demonstrated high factorial correlations and had the best fit across several psychometric model fit indices. Although the mean level K6 score represented mild distress in this sample, the instrument could prove extremely useful for measuring mental health problems among the general population within OPT (and other Arab nations) that experience ongoing violence, poverty, and deprivation [21].

Convergent validity was also assessed using two well-established measures of related psychological problems: anxiety and somatic symptoms. The K6 was highly correlated with both of these measures, providing additional evidence that the instrument may be of value in screening for psychological problems in Arabic-speaking populations. Interestingly, the mean score for somatic symptoms was situated in the high range for the SSS-8. This finding may suggest cultural variations in presentations of mental health symptomology, consistent with previous studies with Arabic samples [37] and the broader, well-documented phenomenon of “idioms of distress” among trauma survivors [38]. As such, it might prove prudent for practitioners and researchers in OPT and other areas with Arabic-speaking populations to use both the K6 and the SSS-8 for clinical assessments.

In interpreting results, several limitations should be kept in mind. The investigation was based on a non-probability sample with relatively high levels of education and employment. Replication studies conducted with larger, population-based samples of Arabic-speaking participants could improve generalizability. Furthermore, the analysis was based on a one-time administration of a single version of the survey, preventing test-retest evaluation or comparison of alternate versions of the same measures. Finally, cognitive interviewing was not used in the translation efforts [39]. Use of this technique can enhance confidence that translated measures are understood as intended by researchers.

To our knowledge, this was the first psychometric study of translated versions of the Kessler Psychological Distress Scale in OPT, an area of the world marked with exposure to ongoing, persistent, and cumulative traumatic stressors. Findings could be relevant for scholars, social workers, and health practitioners working in Arabic-speaking parts of the world, including Middle Eastern nations facing chronic violence and unrest such as Iraq, Syria, Egypt, and Libya. Future research is vital in order to refine our ability to detect and identify vulnerable individuals whose first language is Arabic and who are in need of psychological support services.

## Conclusion

Findings suggest that the translated, two-factor Kessler 6 has good factorial structure and is a reliable and valid instrument to measure psychological distress among Arabic-speaking people in the Occupied Palestinian Territories.

## Abbreviations

AIC: Akaike information criteria
BIC: Bayesian information criteria
CFI: comparative fit index
GAD-7: Generalized Anxiety Disorder
K10: Kessler Psychological Distress Scale (ten-item version)
K6: Kessler Psychological Distress Scale (six-item version)
ML: maximum likelihood estimation
MoSA: Ministry of Social Affairs
MoSD: Ministry of Social Development
OPT: Occupied Palestinian Territories
RMSEA: root mean square error of approximation
SD: standardized deviation.
SPSS: Statistical Package for the Social Sciences
SRMR: standardized root mean square residual
SSS-8: Somatic Symptoms Scale
UNDP: United Nations Development Program
